# Sharing Sensor Data with SensorSA and Cascading Sensor Observation Service

**DOI:** 10.3390/s90705493

**Published:** 2009-07-10

**Authors:** Denis Havlik, Thomas Bleier, Gerald Schimak

**Affiliations:** Austrian Institute of Technology, Donau-City Straße 1, A1220 Vienna, Austria; E-Mails: thomas.bleier@ait.ac.at (T.B.); gerald.schimak@ait.ac.at (G.S.)

**Keywords:** Sensor Observation Service (SOS), cascading SOS, Open Geospatial Consortium (OGC), OGC Sensor Web Enablement (OGC-SWE), Global Earth Observation System of Systems (GEOSS), Global Monitoring for Environment and Security (GMES), SANY IP, ORCHESTRA, data acquisition, UWEDAT, service oriented architecture, events driven architecture

## Abstract

The SANY IP consortium (http://www.sany-ip.eu) has recently developed several interesting service prototypes that extend the usability of the Open Geospatial Consortium “Sensor Web Enablement” (OGC SWE) architecture. One such service prototype, developed by the Austrian Research Centers, is the “cascading SOS” (SOS-X). SOS-X is a client to the underlying OGC Sensor Observation service(s) (SOS). It provides alternative access routes to users (or services) interested in accessing data. In addition to a simple cascading, SOS-X can re-format, re-organize, and merge data from several sources into a single SOS offering. Thanks to the built-in “Formula 3” prototype, a kind of time series library, SOS-X will be enabled to derive new data sets on the fly executing arbitrary algebraic operations on one or more data input streams. This article will discuss the SOS-X development status (focusing at end of 2008), further development agenda in year 2009, and possibilities for using the SOS-X outside of the SANY IP.

## 1. Introduction

Our strategies to conserve and develop the environment in a favourable way often depend on detailed knowledge, not only about single observable properties, but about whole ecosystems, their parts, relationships, and dependencies. Building such know-how was often a tedious task, as data acquisition has traditionally been expensive and data integration is a labour-intensive task of conversions and transformations, often implying information losses.

The importance of the seamless information exchange across administrative and domain borders has been well understood at the European level, leading to increasingly more demanding initiatives and directives. Some prominent examples include:

- “Water Framework Directive” (WFD) [1] demands exchange of water-related information and water management at the river basin level;
- “Infrastructure for Spatial Information in the European Community” (INSPIRE) directive [2] demands seamless exchange of all geo-referenced environmental information through spatial information services.
- “Global Monitoring for Environment and Security” (GMES), [3] is the European contribution to worldwide monitoring and management of our planet Earth and the European contribution to the Group on Earth Observation (GEO) and its implementation plan of an integrated Global Earth Observation System of Systems (GEOSS) [4].

In parallel to the legislative and organisational work, the European Union has invested considerable resources in developing the infostructure capable of answering the GEOSS/GMES challenges. One of the most prominent relevant European research projects, ORCHESTRA IP [5] came to following conclusions:

1. Imposing a single technical infrastructure to all environmental systems is not feasible due to high costs of transition and mismatch between short-lived technology and long-lived environmental monitoring.
2. In addition, defining a single information model for all types of environmental information may not only be difficult and prohibitively expensive, but probably even impossible, because the appropriate data model depends on the purpose for which the information has been published.

Consequently, the ORCHESTRA IP defined the “Reference Model for Orchestra Architecture” (RM-OA) [6] and associated services in form of technology-independent specifications and rules for mapping these specifications to different technology platforms. Sensor Service Architecture (SensorSA) [7] developed by “Sensors Anywhere” Integrated Project (SANY IP) [8] extends the RM-OA through inclusion of the sensor- and sensor-network specific services and event-driven processing.

This article concentrates on prototype implementation of the Cascading SOS (SOS-X) service prototype – a middleware service capable of bridging the technical and domain-induced gaps between information providers and information consumers.

### 1.1. SensorSA’s Inheritance

The Sensor Service Architecture (SensorSA) is a service-oriented architecture (SOA) with elements of an event driven architecture (EDA) and a particular focus on the access, management and processing of information provided by sensors and sensor networks.

SensorSA extends the “Reference Model for ORCHESTRA Architecture” (RM-OA) [6] in the area of in-situ monitoring through (1) inclusion of the OGC “Sensor Web Enablement” service specifications (OGC SWE; see http://www.opengeospatial.org/projects/groups/sensorweb) and (2) definitions of the data models and interaction patterns required for in-situ environmental monitoring. In addition, the SensorSA introduces the elements of Events Driven Architecture (EDA) to RM-OA and the SensorSA implementation architecture explicitly allows simultaneous use of SOAP, REST-full and standard OGC web services.

Sensor Web Enablement (OGC SWE) is a suite of standardized web-service interfaces and XML schemas that allow live integration of heterogeneous sensor webs into an information infrastructure.

RM-OA, which has been accepted as a “Best Practice” by Open Geospatial Consortium (OGC) describes an abstract architecture for environmental risk management, a methodology for mapping this abstract architecture onto the technical platform of choice, and a “system of systems” integration concept as way to achieve interoperability on a technical level.

At the level of information exchange, RM-OA demands self-description of all resources [9]. In particular, the RM-OA states that: “all meta-information MUST be provided at least in a form suitable for interpretation by humans”; “syntactic meta-information MUST also be provided in a form suitable for interpretation by machines”; and “providing semantic meta-information in a form suitable for interpretation by machines (e.g., by means of an ontology) is highly encouraged”.

### 1.2. Cascading SOS User Requirements

Development of the cascading SOS (SOS-X) started as a direct consequence of the request for “sending data on a DVD” issued by SANY partners responsible for data fusion and modelling at the early project phase (Q2 2007). A close analysis of their motives revealed following issues:

1. Models and fusion engines have no knowledge of the SensorSA and expect the data to be provided in a pre-defined file format.
2. The 52 North implementation of the Sensor Observation Service (SOS) [10] we used at that time did not provide mechanisms for handling large data sets.
3. The SOS standard allows great freedom for data modellers. As a result, the SOS data sources deployed by SANY partners diverged, thus hampering the development of the SOS clients.
4. Finally, the SWE standards suite does not define the domain-specific meta-information. Unsurprisingly, the meta-information definitions used by SANY partners turned out to be incompatible, even within a single environmental domain.
5. In the meantime a fifth issue joined the initial quartet: newly developed SOS servers, which follow the v1 of the SOS specification are incompatible with SOS clients that have been initially written with v0.31 of the specification in mind.

The first issue has been solved through encapsulation methods (e.g., of the fusion or modelling engine) in a SensorSA compliant envelope. Today, the modelling and fusion engines deployed in SANY read their input directly from an SOS server. In addition, the OGC Web Processing Service [11] is used to control the processing, and the processed output can be accessed over SOS interface.

Due to the reasons mentioned in introduction, full standardization of data models, network technologies and service interfaces is unlikely in the GEOSS/GMES context. Therefore we decided to develop a service that will ease the pain of software developers and network integrators by mediating between GMES data users and data providers.

## 2. Cascading SOS Use Cases

**Figure 1.**
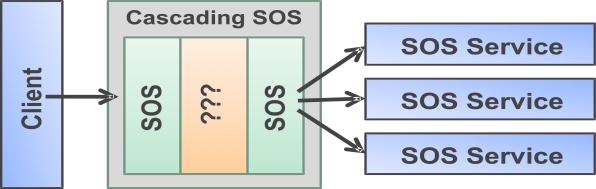
Cascading SOS.

Cascading Sensor Observation Service (SOS-X) is a client to the underlying SOS service(s) and provides alternative access routes to users (or services) interested in accessing data (Figure 1).

In its simplest form, SOS-X provides an alternative route to accessing the data offered by underlying SOS service with no changes to the information model. However, the real power of this service lies in its capability to optimize the data flow, manipulate the meta-information, and to (pre-)process information before re-publishing. The capabilities of the SOS-X are best understood on the basis of use cases (UC), described in the following sections.

### 2.1. UC1: Publishing

**Figure 2.**
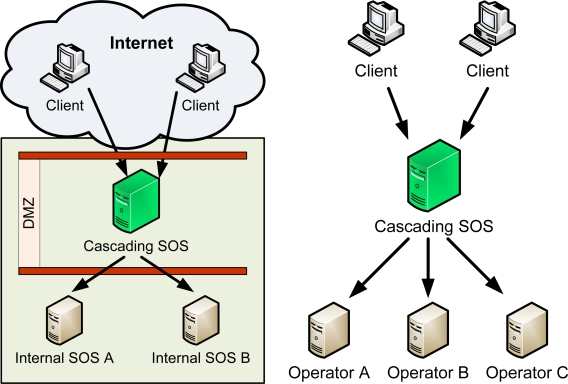
From left to right: (a) UC1 “data publishing”; (b) UC2 “custom view to data”.

In this use case, the cascading SOS is situated in a demilitarized zone (DMZ) and used to publish a subset of internally available data to the outside world (Figure 2a). This mode of operation allows clean separation of the data into “confidential/internal” and “public” part, without the need to replicate the data. In addition, the SOS-X can be instructed to customize the view to the data before re-publishing (UC2, see Figure 2b).

A major security disadvantage of this solution is the need to allow the access to multiple internal servers from the demilitarized zone. For non-transient data, this security risk could be eliminated by pushing the data to SOS-X and serving it from its cache. This requires support for event-handling and permanent data storage (UC4, Section 2.4).

### 2.2. UC2: Custom View to Data

In this use case, the cascading SOS is used to provide a single point of access to data from several sources (Figure 2b). The data served by SOS-X itself remains unchanged in this process, but the meta-information and the way data is presented to the users may be altered in the process. This is similar in spirit of ORCHESTRA Translating Feature Access Service (FAS-X) [12], but very different in its implementation. While the FAS-X directly manipulates the XML, the SOS-X translates all incoming data and meta-information into an internal data model.

This architectural decision has one very useful implication: SOS-X can be easily extended to use other sources of information instead of, or in parallel with those available using a Sensor Observation Service interface.

### 2.3. UC3: Protocol Transducer

Environmental monitoring systems are often used for decades, but the technology often changes very rapidly. As already mentioned in Section 1.2, the SWE specifications were still immature at the time we started the project, and changed in a way that renders the initially used services incompatible with the new developments. This kind of service interfaces incompatibility is a rule rather than exception in the real world.

Cascading SOS can be used to bridge the technology gap between information providers and users (Figure 2a). As already mentioned in Section 2.2, the SOS-X implementation architecture is modular, and (back-end) clients can be written for various legacy services. In addition, the front-end interface is also implemented in a way that allows easy exchange or even simultaneous access to data over several interfaces.

### 2.4. UC4: SOS Proxy

Information offered by a SOS often contains a large archive of historic data. For example, an air quality monitoring system may contain more than twenty years of archived information. The archive slowly grows with time (e.g., one new value per sensor every 30 minutes in the case of an air quality monitoring system), and the archived data rarely (if ever) changes.

Archived data is indispensable, e.g., for trend analysis, and training of models and data fusion engines, but fetching it over slow internet connections may be a very time consuming activity. In addition, most SOS server implementations lack support for serving large data sets. The Sensor Observation Service specification in principle allows panning, binary payloads and out of band data delivery. However, neither of these mechanisms is compulsory, and none have been widely used so far.

**Figure 3.**
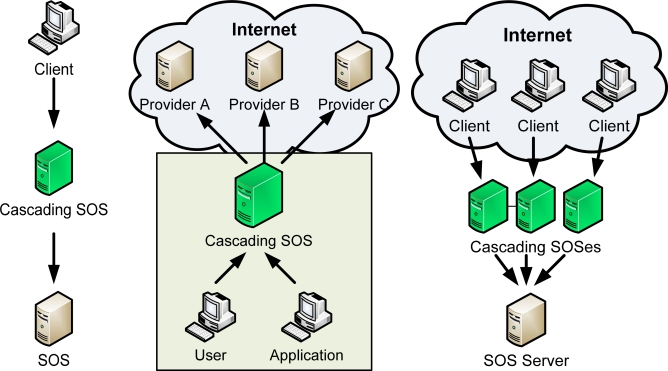
From left to right: (a) UC3 “Protocol Transducer”; (b) UC4 “SOS Proxy”; (c) UC5 “Load Balancing”.

SOS-X residing on a users LAN could greatly improve the quality of service by pre-fetching and caching the data (Figure 3b). In addition, the SOS-X could implement advanced mechanisms for serving large data sets.

### 2.5. UC5: Simple Load Balancing

In emergency situations, the number of requests for information may rise far beyond the average needed server capacity. In addition, most requests concern only a tiny subset of the data. This, and the stateless nature of the SOS service assures that scaling-out is a good answer for emergency overloads.

SOS-X allows a very simple mechanism for scaling out: the original server is moved to the background, and replaced by a group of SOS-X servers. Each of the SOS-X servers is configured to act as exact replica of the original system, and a load balancer assures that the load is evenly distributed over all servers. Thanks to SOS-X caching mechanism, most of the requests can be handled by one of the SOS-X servers, without the need to consult the original SOS service.

### 2.6. UC6: Value Added SOS

**Figure 4.**
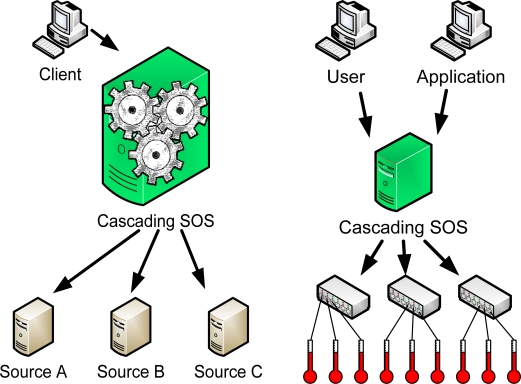
From left to right: (a) UC6 “Value Added SOS”; (b) UC2 “Sensor Data Store”.

This use case is an advanced version of the UC1 and UC2. SOS-X features a built-in mechanism for performing arbitrary algebra operations on time series (Figure 4a). The algebra operations are performed by the “Formula 3” (F3) engine, which is developed in parallel with the SOS-X, and may in the future even be made available as a stand-alone software independent from cascading SOS. Unfortunately, no publicly available documentation of F3 has been published so far. Typical (pre-) processing tasks that can be performed by SOS-X include:

- units conversions,
- building of indicators and
- re-sampling of data.

### 2.7. UC7: Sensor Data Store

The “Sensor Data Store” use case can be seen as an advanced version of the “SOS Proxy” (Figure 4b). In use cases 1 to 6, we presume that the cascading SOS does not need to keep a local copy of the original or derived data, except for performance reasons. This implies that:

- the original data will be available on the source server in the future and
- all changes to original data must be reflected in the SOS-X.

These two assumptions are correct in most cases. However, two very important cases exist when these assumptions fail, and the SOS-X needs to manage the data on its own:

1. First, the SOS-X could be used as a back-end to a decisions support system. Whenever a decision is made, a snapshot of the data the decision was based on needs to be saved for reference purpose. This could be (and often is) done by the decisions support application, but keeping the snapshot of the data in SOS-X would allow more flexibility (e.g., comparing the performance of different algorithms).
2. Second, some SOS servers may have a limited storage capacity, or even only serve the current sensor value(s). While the information obtained from such servers remains perfectly valid, it may not be available on the original server the next time we need it.

## 3. Development Status and Outlook

The development of SOS-X started in Q1 2008. In the meantime, a first working prototype has been presented to potential users on the SANY SP4 (Air Quality) demonstration event (November 4th, 2008 in Vienna, Austria). The software is written in Java (J2EE) and uses 52 North’s implementation of the SOS specification as it’s front-end.

The seven use cases introduced in Section 2 should be seen as simplified development specifications, and do not reflect the current development status. The current prototype is capable of demonstrating the use cases 1, 2, 3 and 6 in a controlled environment, but it does not fully support all optional features of the SOS specifications (e.g., space filtering has not been implemented yet) and is definitely not ready for being used in a productive environment. As a proof of concept, the current SOS-X prototype can currently perform following tasks:

1. Provide custom views to data originating from other SOS servers and from a proprietary UWEDAT system [13]
2. alter meta-information provided by the source system and add missing meta-information to the data
3. perform the calculation of a moving average on time series
4. translate between SOS 0.31 and SOS 1.0 protocols

Although not complete, the current prototype is very encouraging, as it confirms the validity of our initial assumptions. Further financing of the development is assured until the end of 2009 through SANY IP, and our immediate development agenda foresees following tasks:

- bug fixing and stabilization of the existing code; clean separation of the 52 North SOS code from the SOS-X code developed by ARC; assuring compliance with SOS specifications.
- permanent data storage and a simple mechanism for pre-fetching and caching of data; define a SensorML representation of meta-information to indicate the “time to live” and frequency of updates.
- (simple) mechanism for handling large data sets; improve F3; add preliminary support for event processing; automatic recognition of meta-information relevant for caching; demonstration of all seven Use Cases;
- consolidation of the development done so far; concentrate on performance and bug fixing; if possible add support for SOS over SOAP, user management, authorization and authentication.

This development agenda aims to establish solid software architecture and to maximize the usability of SOS-X in various SANY demonstrations. Post-SANY development agenda will be driven by user requirements and needs of the future ARC research projects. Some of the envisaged post-SANY topics include development of the user-friendly client for SOS-X configuration; rich set of pre-defined F3 functions; advanced SOS data models; advanced caching mechanisms; and developing the clients for additional legacy systems.

## 4. Conclusions

Cascading SOS is a very promising concept, with the potential of becoming a very important infrastructure building block for the *in-situ* sector of GMES, GEOSS, INSPIRE and other large in-situ environmental monitoring networks. The results of the first development cycle are encouraging and no conceptual problems have been discovered so far. However, the software is still in an early stage, and the usability of the final product will depend on the performance of the SOS-caching method(s).

In order to improve the visibility of the project, and build communities interested in further development beyond the end of the SANY IP, ARC decided to publish SOS-X under Open Source (GPL license). All information concerning the development status and instructions for downloading and installing the software is available on the SANY-IP web site (http://sany-ip.eu/results/sos_x).
